# Biosynthesis and signal transduction of plant growth regulators and their effects on bioactive compound production in *Salvia miltiorrhiza* (Danshen)

**DOI:** 10.1186/s13020-024-00971-5

**Published:** 2024-07-24

**Authors:** Heqin Li, Xuwen Jiang, Kiyoshi Mashiguchi, Shinjiro Yamaguchi, Shanfa Lu

**Affiliations:** 1 College of Agronomy, Qingdao Agricultural University, No. 700 Changcheng Road, Chengyang District, Qingdao, 266109 Shandong People’s Republic of China; 2 Institute for Chemical Research, Kyoto University, Gokasho, Uji, Kyoto 611-0011 Japan; 3 Institute of Medicinal Plant Development, Chinese Academy of Medical Sciences and Peking Union Medical College, No. 151 Malianwa North Road, Haidian District, Beijing, 100193 People’s Republic of China; 4 Shandong Bairuijia Food Co., Ltd, No. 8008, Yi Road, Laizhou, Yantai, 261400 Shandong People’s Republic of China

**Keywords:** Plant growth regulators, Phenolic acids, Tanshinones, *Salvia miltiorrhiza*, Danshen

## Abstract

Plant growth regulators (PGRs) are involved in multiple aspects of plant life, including plant growth, development, and response to environmental stimuli. They are also vital for the formation of secondary metabolites in various plants. *Salvia miltiorrhiza* is a famous herbal medicine and has been used commonly for > 2000 years in China, as well as widely used in many other countries. *S. miltiorrhiza* is extensively used to treat cardiovascular and cerebrovascular diseases in clinical practices and has specific merit against various diseases. Owing to its outstanding medicinal and commercial potential, *S. miltiorrhiza* has been extensively investigated as an ideal model system for medicinal plant biology. Tanshinones and phenolic acids are primary pharmacological constituents of *S. miltiorrhiza*. As the growing market for *S. miltiorrhiza*, the enhancement of its bioactive compounds has become a research hotspot. *S. miltiorrhiza* exhibits a significant response to various PGRs in the production of phenolic acids and tanshinones. Here, we briefly review the biosynthesis and signal transduction of PGRs in plants. The effects and mechanisms of PGRs on bioactive compound production in *S. miltiorrhiza* are systematically summarized and future research is discussed. This article provides a scientific basis for further research, cultivation, and metabolic engineering in *S. miltiorrhiza*.

## Introduction

Plant growth regulators, often known as PGRs, are artificially produced or naturally occurring substances that influence the development and metabolism of higher plants, even at low concentrations. In general, PGRs include natural plant hormones (also known as phytohormones) and their synthetic analogs, polyamines (PAs), nitric oxide (NO), and other compounds [1–3]. Auxins (AUXs), cytokinins (CKs), gibberellins (GAs), abscisic acid (ABA), and ethylene (ET) are classified as the classic phytohormones. Recently, salicylic acid (SA), brassinosteroids (BRs), and jasmonates (JAs) have been identified. Strigolactones (SLs) and karrikins (KARs) or KAR INSENSITIVE2 (KAI2) ligands (KLs) are two promising classes of PGRs that have garnered a great deal of attention due to their multiple biological effects on plant growth, development, and stress adaptation [4–6]. Melatonin is also regarded as an intriguing PGR. It has growth-promoting and anti-stress properties and is also capable of regulating the activities of various other plant hormones [7, 8]. However, the classification of NO has not yet been confirmed [9, 10].

**Fig. 1:**
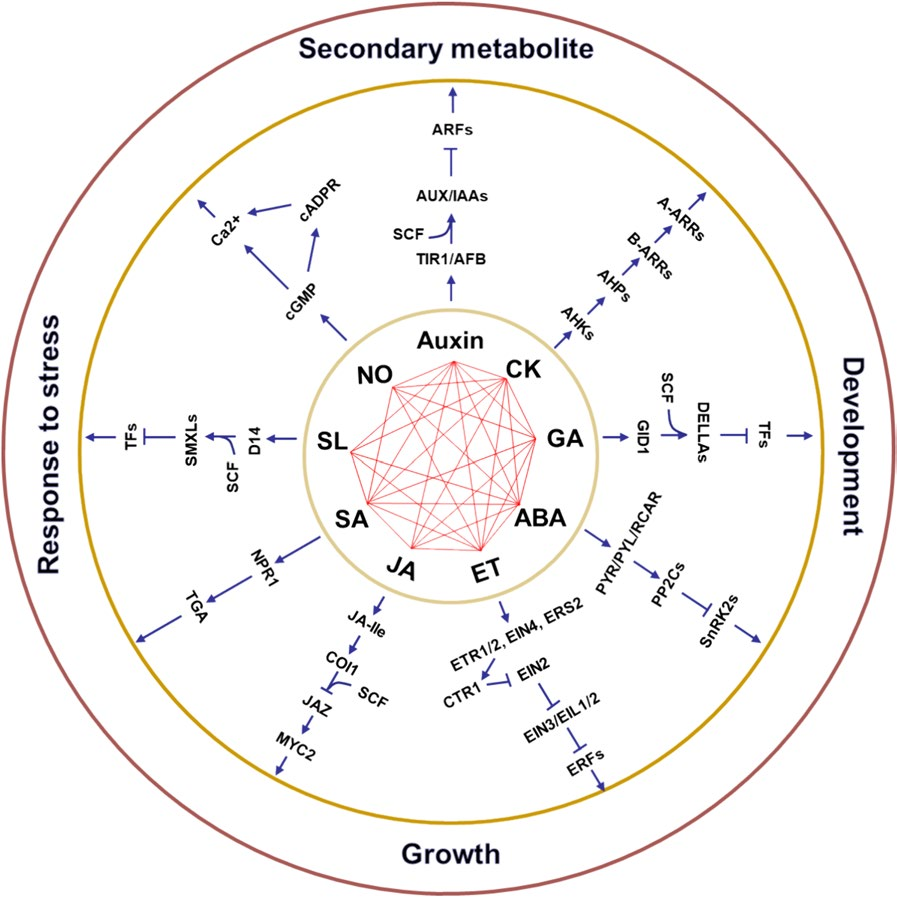
The signal transduction and role of plant growth regulators. CK: cytokinin, GA: gibberellin, ABA: abscisic acid, SA: salicylic acid, ET: ethylene, JA: jasmonic acid, SL: strigolactone, NO: nitric oxide, TIR1/AFB: transport inhibitor response1/Auxin signaling F-box, AUX/IAA: auxin/indole-3-acetic acid, ARF: auxin response factor, AHK: arabidopsis histidine kinase, AHP: arabidopsis phosphotransfer protein, B-ARR: type-B response regulator, A-ARR: type-A response regulator, GID1: gibberellin insensitive dwarf1, DELLAs: DELLA proteins, TF: transcription factor, SCF: Skp-Cullin-F-box, PYR/PYL/RCAR: pyrabactin resistance/pyrabactin resistance-like/regulatory components of the abscisic acid receptor, PP2C: phosphatase clade-A type-2C protein phosphatase, SnRK2: SNF1-related protein kinases 2, NPR1: nonexpressor of PR1, TGA: TGACG motif-binding factor, ETR1/2: ethylene receptor 1/2, EIN2: ethylene insensitive 2, EIN3: ethylene insensitive 3, EIL1/2: EIN3-like1/2, ERF: ethylene responsive factor, JA-Ile: jasmonoyl-isoleucine, COI1: coronatine insensitive1, JAZ: jasmonate ZIM-domain, D14: DWARF14, SMXL: suppressor of MAX2 1-like, cGMP: cyclic guanosine monophosphate, cADPR: cyclic ADP-ribose. Red line in the center of circle represents the crosstalk of PGRs. Arrows and bars at the end of each line show positive and negative regulations, respectively

Collectively, these PGRs regulate all aspects of plant life via a complex regulatory network, from seed germination to senescence, under both normal and stress conditions. In some cases, a single PGR may affect a variety of cellular and developmental processes under specific conditions, whereas in others, multiple PGRs may be involved in regulating a single function [11, 12] (Fig. 1). Moreover, AUXs regulate seed germination and the development of roots, shoots, and fruits, either independently or through interactions with other PGRs, such as ABA, CKs, ET, GAs, JAs, SA, BRs, and SLs [13, 14]. CKs, alone or in coordination with other PGRs (such as ABA, ET, AUXs, SLs, and GAs), regulate leaf growth, vascular patterning, stomatal production, nutrient metabolism, and plant adaptation to abiotic and biotic stimuli [15–19]. GAs regulate multiple developmental processes of plants, such as seed germination, stem elongation, leaf expansion, and flower and fruit development [20–23], either independently or through their interactions with ABA, CKs, JAs, and AUXs [24]. High levels of ABA and its metabolites appear to be associated with dormancy maintenance and seed development in certain seeds, buds, and fruits. ABA regulates various physiological processes in response to abiotic stimuli by crosstalk with GAs, MT, CKs, AUXs, SA, JAs, ET, BRs, and SLs [25]. ET has multiple roles in plants, including fruit ripening, growth, senescence, seed germination, flowering, and responses to different environmental stimuli [26]. In addition, the coordination of ET and AUXs along with ABA, GAs, CKs, JAs, and BRs also contribute to primary root formation [27].

The complexity of the crosstalk between JAs and other phytohormone signals (GAs, CKs, AUXs, SA, and ABA) during plant development and stress adaptation has been demonstrated [28]. In addition, interactions between SA and ABA, JAs, BRs, CKs, GAs, ET, NO, etc. have been reported [29]. SA is regarded as a well-known hormone found in plants that is essential for basic immunity and systemic acquisition of resistance [30]. In plant environmental stress response and development, the activation of biosynthesis of a diverse set of secondary metabolites is one of the primary and most noticeable responses to JAs [31]. In recent years, SLs are a new kind of hormones discovered in plants with a variety of roles including seed germination, adventitious rooting, internode elongation, secondary growth, shoot branching, leaf development, tolerance and resistance to abiotic and biotic stimuli, and secondary metabolism [5, 32–36]. SLs and their crosstalk with other PGRs, such as AUXs, CKs, ABA, GAs, ET, SA, and JAs have also been studied [37]. NO also regulates plant growth, development, and stress adaptation by means of a complicated network that includes crosstalk with other PGRs, such as ABA, GAs, ET, AUXs, and CKs [3, 38, 39]. Additionally, these PGRs also assisting in the synthesis of secondary metabolites, including phenolics [33, 40–46], alkaloids [47], and terpenoids [48, 49].

**Fig. 2:**
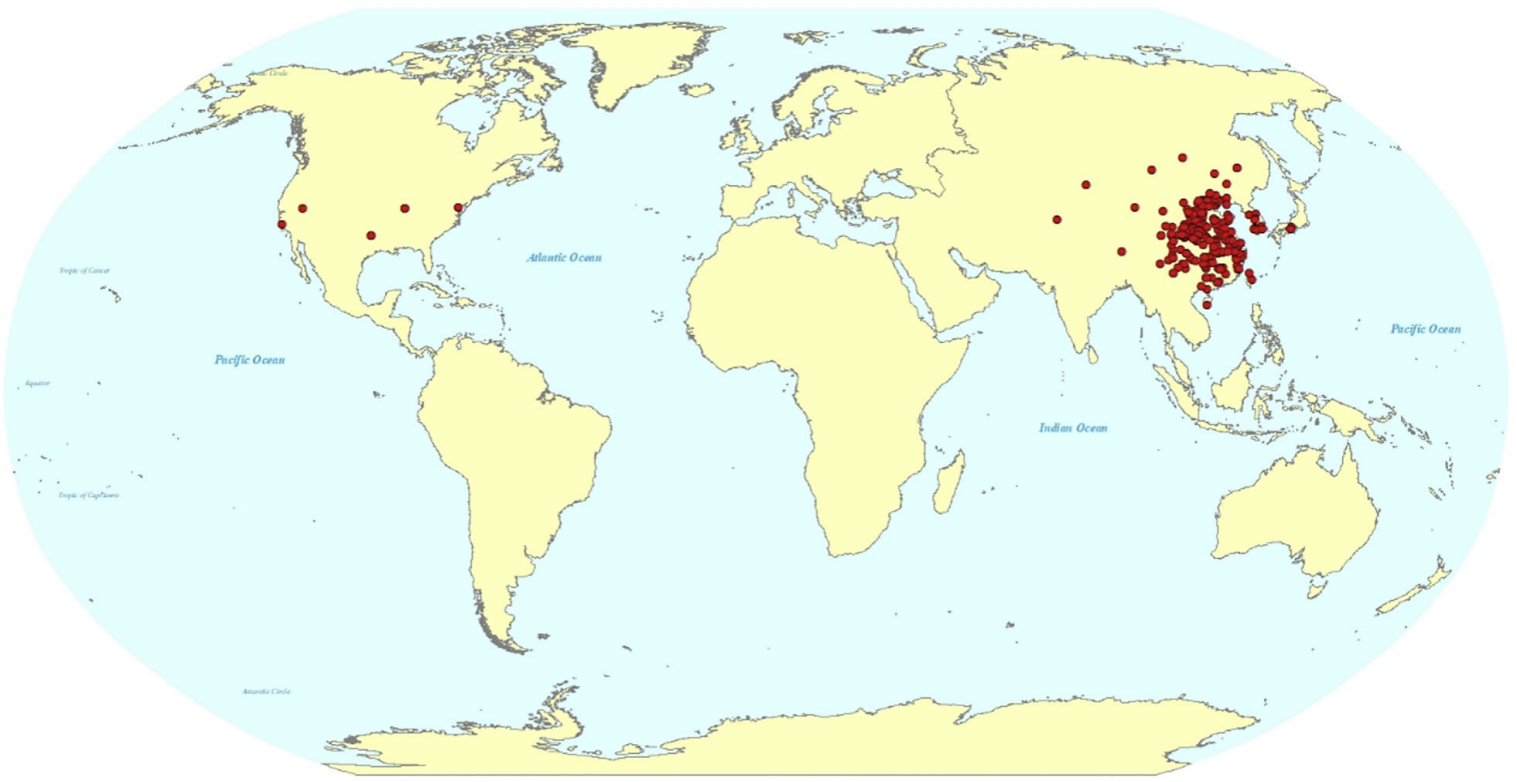
The geographical distribution of *S. miltiorrhiza*. Data were obtained from the Global Biodiversity Information Facility (https://www.gbif.org/)

**Fig. 3:**
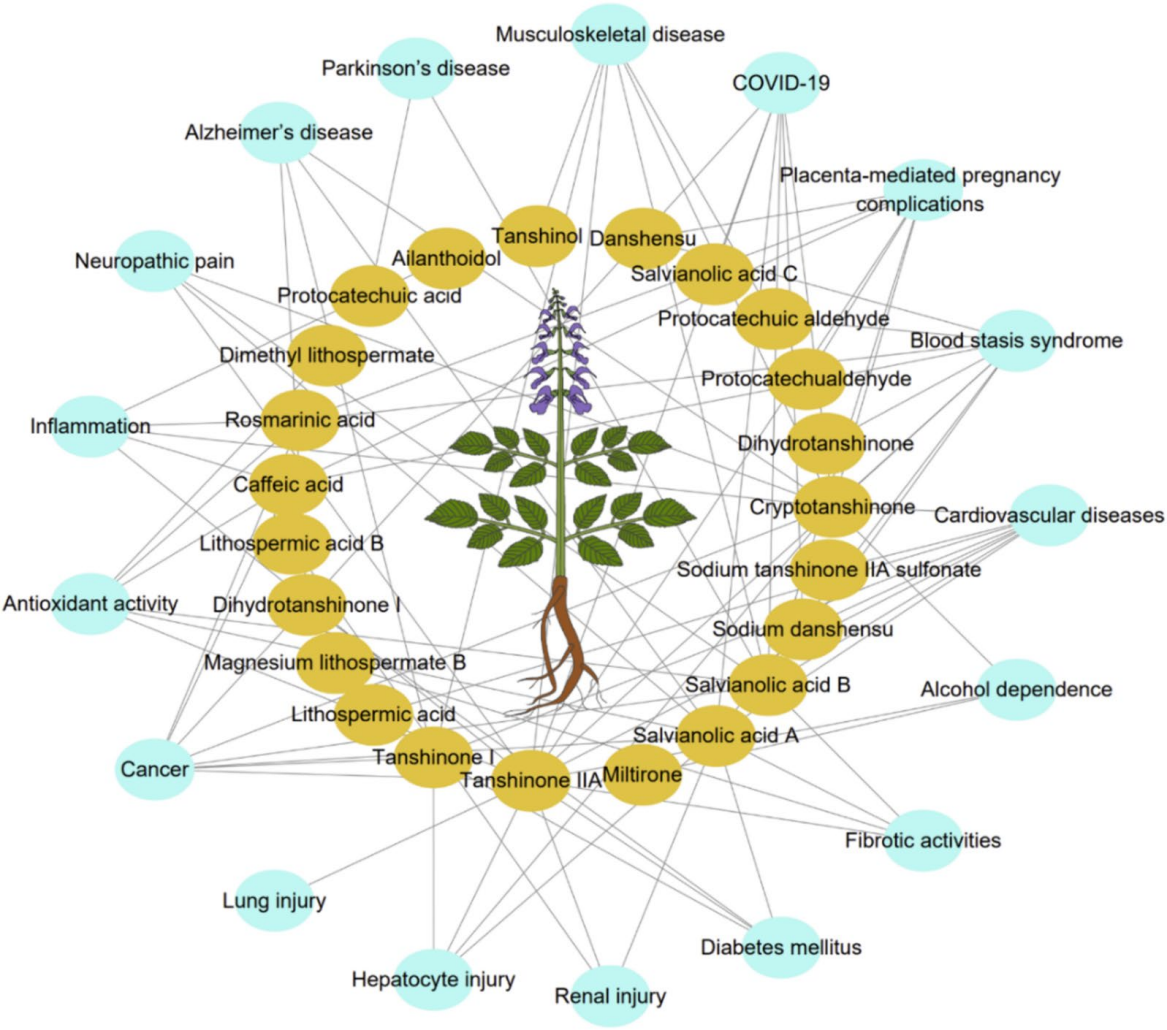
The pharmacological functions of the active compounds of *S. miltiorrhiza*. The networks were visualized using Cytoscape software (v. 3.10.2) (https://cytoscape.org/)

In the genus *Salvia* of the family Lamiaceae, *Salvia miltiorrhiza* Bunge is one of the most famous species. In China, it is frequently referred to as Danshen, red sage, or Chinese sage. It is a perennial herb that originated in China and is also found in Mongolia, Korea, Japan, America, and New Zealand (Fig. 2). Usually, wild *S. miltiorrhiza* grows in sunny hillside grass, ditch edge, roadside or forest edge. In traditional Chinese medicine, *S. miltiorrhiza* has been used for > 2000 years because of its excellent medicinal properties [50]. Its main effects were to increase blood flow, eliminate blood stasis, and induce mental calmness long before [50, 51]. Now, people use it to treat a range of illnesses, including cardiovascular diseases [51–59], cerebrovascular disease [51, 52, 57, 58, 60], Alzheimer’s disease [52, 57, 61, 62], inflammation [52, 57, 60, 63], Parkinson’s disease [52, 57, 63], renal deficiency [64], hepatocirrhosis [65], cancer [52, 57, 58, 60, 66–68], musculoskeletal diseases [69], diabetes and related diseases [52, 57, 70, 71], blood stasis syndrome [72], and placenta-mediated pregnancy complications [73], either by simple preparations or as a component of traditional Chinese patented medicines. Recently, its potential as a treatment for the virus that causes coronavirus disease 2019 (COVID-19), has drawn more attention [74–79] (Fig. 3). To date, China, Japan, the United States, and many European countries have all used *S. miltiorrhiza* to treat various diseases [54]. The quality standards for *S. miltiorrhiza* are strictly set in the Chinese Pharmacopoeia, Japanese Pharmacopoeia, United States Pharmacopoeia, and European Pharmacopoeia (Table 1). Comparative analysis of these pharmacopoeia, the quality standards of *S. miltiorrhiza* in the United States Pharmacopoeia are stricter, while in the Japanese Pharmacopoeia are slightly looser, and the Chinese Pharmacopoeia is relatively close to the United States Pharmacopoeia. Moreover, it is believed that *S. miltiorrhiza* is an ideal model system for studying the biology of medicinal plants due to its short life cycle, high vitality, effective transgenic technology, quiet small genome, and a low number of chromosomes [80, 81]. Therefore, *S. miltiorrhiza* holds significant importance in the economic, academic, and medicinal fields [81].

**Table 1:** The quality standards of *S. miltiorrhiza* in Chinese Pharmacopoeia, Japanese Pharmacopoeia, the United States Pharmacopoeia, European Pharmacopoeia

Item	European Pharmacopoeia (version 11.5)	Japanese Pharmacopoeia (version 18.0)	Chinese Pharmacopoeia (version 2020)	The United States Pharmacopoeia (version 1.0)
Material origin	Synthetic	Dried roots	Dried roots and rhizomes	Dried roots and rhizomes
Loss on drying		≤ 16%	≤ 13.0%	≤ 13.0%
Total ash		≤ 7.5%	≤ 10.0%	≤ 4.0%
Acid-insoluble ash		≤ 2.0%	≤ 3.0%	≤ 3.0%
Arsenic		≤ 5 ppm	≤ 2 mg/kg	≤ 2.0 µg/g
Cadmium			≤ 1 mg/kg	≤ 0.3 µg/g
Lead		≤ 10 ppm	≤ 5 mg/kg	≤ 5.0 µg/g
Mercury			≤ 0.2 mg/kg	≤ 0.2 µg/g
Copper			≤ 20 mg/kg	
Alcohol-soluble extractives		≥ 42.0%	≥ 15.0%	≥ 15.0%
Water-soluble extractives			≥ 35.0%	≥ 35.0%
Total tanshinones (cryptotanshinone, tanshinone I, tanshinone IIA)			≥ 0.25%	≥ 0.2%
Tanshinone IIA				≥ 0.1%
Salvianolic acid B	15 mg/20 g CRS		≥ 3.0%	≥ 3.0%
Phenolic acids				Salvianolic acid, lithospermic acid, and rosmarinic acid

**Fig. 4:**
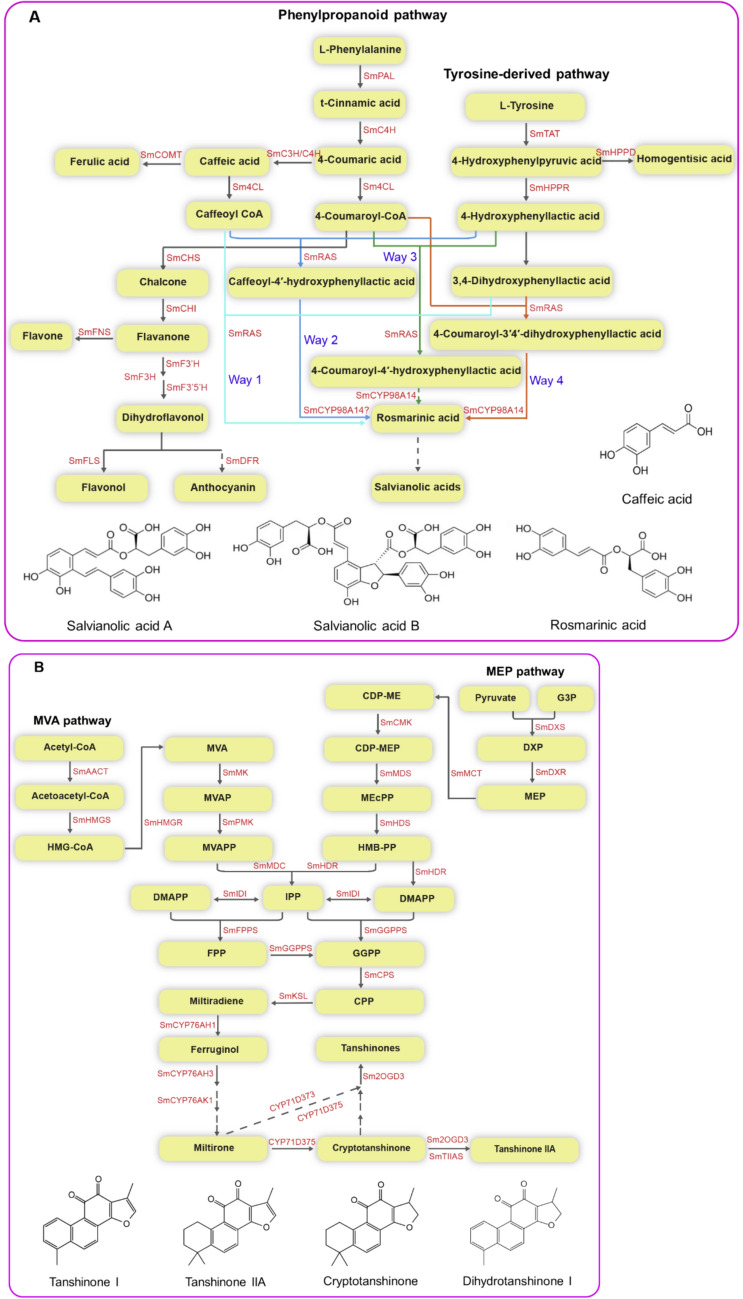
The biosynthetic pathway of secondary metabolites in *S. miltiorrhiza*. SmPAL: phenylalanine ammonia lyase, SmC4H: cinnamate 4-hydroxylase, Sm4CL: 4-coumaroyl CoA ligase, SmTAT: tyrosine aminotransferase, SmHPPR: 4-hydroxyphenylpyruvate reductase, SmHPPD: 4-hydroxyphenylpyruvate dioxygenase, SmRAS: rosmarinic acid synthase, SmC3H: 4-coumaroyl shikimate 3′-hydroxylase/coumarate 3-hydroxylase, SmCOMT: caffeic acid O-methyltransferase, SmCHS: chalcone synthase, SmCHI: chalcone isomerase, SmFNS: flavone synthase, SmF3H: flavanone 3-hydroxylase, SmF3′H: flavonoid 3′-hydroxylase, SmF3′5′H: flavonoid 3′,5′-hydroxylase, SmFLS: flavonol synthase, SmDFR: dihydroflavonol reductase. SmAACT: acetyl-CoA C-acetyltransferase, SmHMGS: hydroxymethylglutaryl-CoA synthase, HMG-CoA: 3-hydroxy-3-methylglutaryl-CoA, SmHMGR: 3-hydroxy-3-methylglutaryl-CoA reductase, MVA: Mevalonate, MVAP: Mevalonate-5-phosphate, SmMK: mevalonate kinase, SmPMK: 5-phosphomevalonate kinase, MVAPP: Mevalonate-5-diphosphate, SmMDC: mevalonate pyrophosphate decarboxylase, SmIDI: isopentenyl pyrophosphate isomerase, DMAPP: dimethylallyl pyrophosphate, IPP: Isopentenyl diphosphate, SmFPPS: farnesyl diphosphate synthase, FPP: Farnesyl diphosphate, G3P: Glyceraldehyde -3-phosphate, SmDXS: 1-deoxy-D -xylulose-5-phosphate synthase, DXP: 1-Deoxy-d-xylulose-5-phosphate, SmDXR: 1-deoxy-d-xylulose 5-phosphate reductoisomerase, MEP: 2-C-methyl-d-erythritol-4-phosphate, SmMCT: 2-C-methyl-d-erythritol 4-phosphate cytidylyltransferase, CDP-ME: 4-(cytidine 5′-diphospho)-2-C-Methyl-d-erythritol, SmCMK: 4-(cytidine 5′-diphospho)-2-C-Methyl-d-erythritol kinase, CDP-MEP: 2-Phospho-4-(cytidine 5′-diphospho)-2-C-methyl-d-erythritol, SmMDS: 2-C-methyl-d-erythritol 2,4-cyclodiphosbphate synthase, MEcPP: 2-C-Methyl-d-erythritol-2,4-cyclodiphosphate, SmHDS: 4-hydroxy-3-methylbut-2-enyl diphosphate synthase, HMB-PP: 4-Hydroxy-3-methylbut-2-enyl diphosphate, SmHDR: 4-hydroxy-3-methylbut-2-enyl diphosphate reductase, SmGGPPS: geranylgeranyl diphosphate synthase, GGPP: Geranylgeranyl diphosphate, SmCPS: copalyl diphosphate synthase, CPP: Copalyl diphosphate, SmKSL: kaurene synthase-like cyclase, Sm2OGD3: 2-oxoglutarate-dependent dioxygenase 3, SmTIIAS: tanshinone IIA synthase. Dashed arrows denote multiple steps. Solid arrows represent single biosynthetic steps. Different color lines represent the different biosynthesis pathways

The dried roots and rhizomes of *S. miltiorrhiza* act as medicinal parts, which are brownish red or brick red in surface color, white inner surface. Until now, over 200 substances have been discovered and isolated from *S. miltiorrhiza* [50]. The primary pharmacological components of this plant are hydrophilic phenolic substances and lipophilic diterpenoid quinones, which are produced through secondary metabolic pathways. Hydrophilic phenolic substances, including rosmarinic acid, caffeic acid, salvianolic acid A, salvianolic acid B and are produced via phenylpropanoid and tyrosine-derived pathways [82, 83] (Fig. 4a). Salvianolic acid B, one of the quality control indicators of *S. miltiorrhiza* in Chinese pharmacopeia and United States Pharmacopoeia, is mainly distributed in the phloem and xylem of *S. miltiorrhiza* roots [84]. Some key enzymes, such as phenylalanine ammonia-lyase (PAL), cinnamate 4-hydroxylase (C4H), 4-coumaroyl CoA ligase (4CL), tyrosine aminotransferase (TAT), *p*-hydroxyphenylpyruvate reductase (HPPR), rosmarinic acid synthase (RAS), and CYP98A14, are involved in the pathways [81, 82]. For rosmarinic acid, although its biosynthetic pathway is still not fully understood, four biosynthetic pathways have been proposed for its formation, including the synthesis of rosmarinic acid from caffeoyl-CoA and 3,4-dihydroxyphenyllactic acid (Way 1), the caffeoyl-4′-hydroxyphenyllactic acid branch (Way 2), the 4-coumaroyl-4′-hydroxyphenyllactic acid branch (Way 3), and the 4-coumaroyl-3′,4′-dihydroxyphenyllactic acid branch (Way 4) [83, 85] (Fig. 4a). Lipophilic diterpenoid quinones, the focus of tanshinone analogues, including dihydrotanshnone I, tanshinone I, tanshinone IIA, and cryptotanshinone, originate from the 2-C-methyl-d-erythritol-4-phosphate (MEP) pathway and/or the mevalonate (MVA) pathway [86, 87] (Fig. 4b). The main processes in the MEP pathway and/or the MVA pathway include isoprene precursor synthesis, direct skeleton precursor production, and tanshinones formation [88]. All terpenes go through the first two processes, but terpenoid synthases and modifying enzymes, which can vary depending on the species, are involved in the latter [88]. According to previous report, tanshinones are mostly present in the periderm [80]. It is generally believed that the root of *S. miltiorrhiza* with deep crimson-colored brown and extensive branches has a higher content of tanshinone. Some key enzymes, including acetyl-CoA C-acetyltransferase (AACT), hydroxymethylglutaryl-CoA reductase (HMGR), 1-deoxy-d-xylulose 5-phosphate synthase (DXS), 1-deoxy-d-xylulose5-phosphate reductoisomerase (DXR), isopentenyl diphosphate (IPP), dimethylallyl diphosphate (DMAPP), geranylgeranyl diphosphate synthase (GGPPS), copalyl diphosphate synthase (CPS), kaurene synthase-like (KSL), cytochrome P450 monooxygenases (CYP76AH1, CYP76AH3, CYP76AK1, CYP71D373, CYP71D375), and 2-oxoglutarate-dependent dioxygenase 3 (2OGD3 or 2-ODD3), along with other unknown enzymes, are associated with the tanshinone biosynthetic pathway [80, 89–95] (Fig. 4b). Additionally, polysaccharides and other secondary metabolites, such as monoterpenes, sesquiterpenes, triterpenes, sterols, flavonoids, anthocyanidins, proanthocyanidins, alkaloids, and quinones, play important roles in medicines [54, 81, 96–100]. A previous study reported that salvinolic acids and tanshinones made up most of the active compounds found in roots, whereas stachyose made up the majority of saccharides; except for tanshinones, salvianolic acids, flavonoids, and triterpenes were found in the leaves, stems, and flowers, and the majority of the saccharides were monosaccharides [101]. Based on traditional Chinese medicine and the active ingredients present, several commercial medicines derived from *S. miltiorrhiza* have been developed in China. They include Danshen injection, Danshensu sodium injection, salvianolic acid B injection, Danhong injection, tanshinone IIA sodium sulfonate injection, salvia polyphenolic acid salt injection, compound Danshen tablets, and compound Danshen dripping pills [71, 102]. In addition, healthcare products such as Danshen flower tea, Danshen leaf tea, and cosmetics contained Danshen extract have also been developed in China. We believe that *S. miltiorrhiza* will have a broader application in food, medicine, cosmetics, healthcare products and other industries in future. Due to its exceptional medicinal and commercial usefulness, *S. miltiorrhiza* has generated significant interest across various fields, including germplasm resources, cultivation, reproduction, biotechnology, and functional genomics [81, 92–94, 103]. The improvement of the bioactive compound content in *S. miltiorrhiza* has become a focus of current study.

In *S. miltiorrhiza*, although there are limited references to the regulatory roles of PGRs on plant growth and development, significant advancements have been made in understanding the regulatory roles of PGRs in the secondary metabolite accumulation. In this article, to better understand the mechanism of action of PGRs on active compound production of *S. miltiorrhiza*, the biosynthesis and signal transduction pathways of PGRs in plants are briefly reviewed. Then, the effects of PGRs on bioactive compound production, mainly phenolic acids and tanshinones, and the underlying mechanisms in *S. miltiorrhiza*, are systematically summarized. Future research perspectives are also discussed. This article provides important implications for further research into the function and regulatory mechanisms of PGRs in the active compound production, field cultivation and metabolic engineering of* S. miltiorrhiza*.

## Biosynthesis and signal transduction of PGRs in plants

### Auxins

AUX was the first hormone to be detected in plants. Indole-3-acetic acid (IAA), indole-3-butyric acid (IBA), 4-chloroindole-3-acetic acid (4-Cl-IAA), and phenylacetic acid (PAA) are endogenous AUXs identified in plants, whereas 1-naphthaleneacetic acid (NAA), 2,4-dichlorophenoxyacetic acid (2,4-d), 2,4,5-trichlorophenoxyacetic acid (2,4,5-T), 4-amino-3,5,6-trichloropicolinic acid (tordon or picloram), and 3,6-dichloro-2-methoxybenzoic acid (dicamba) are endogenous AUX analogs [104]. Of the known AUXs, IAA is the most active form in many plants [105]. Recently, it was reported that the primary process for producing IAA is the Trp-dependent pathway, while the Trp-independent pathway is not a major route [106]. AUXs are primarily formed in developing tissues and transported to specific locations based on polarity to influence the various aspects of plant life activities [13]. Plant cells respond to auxins by means of the transport inhibitor response 1/auxin signaling F-box (TIR1/AFB) auxin coreceptor, auxin/indole-3-acetic acid (AUX/IAA) transcriptional repressor, and auxin response factor (ARF) transcription factor signaling pathway, which are all located in the nucleus of *Arabidopsis* [107, 108] (Fig. 1). AUX/IAA and ARF are important controller of AUX signal transduction [109].

### Cytokinins

**Fig. 5:**
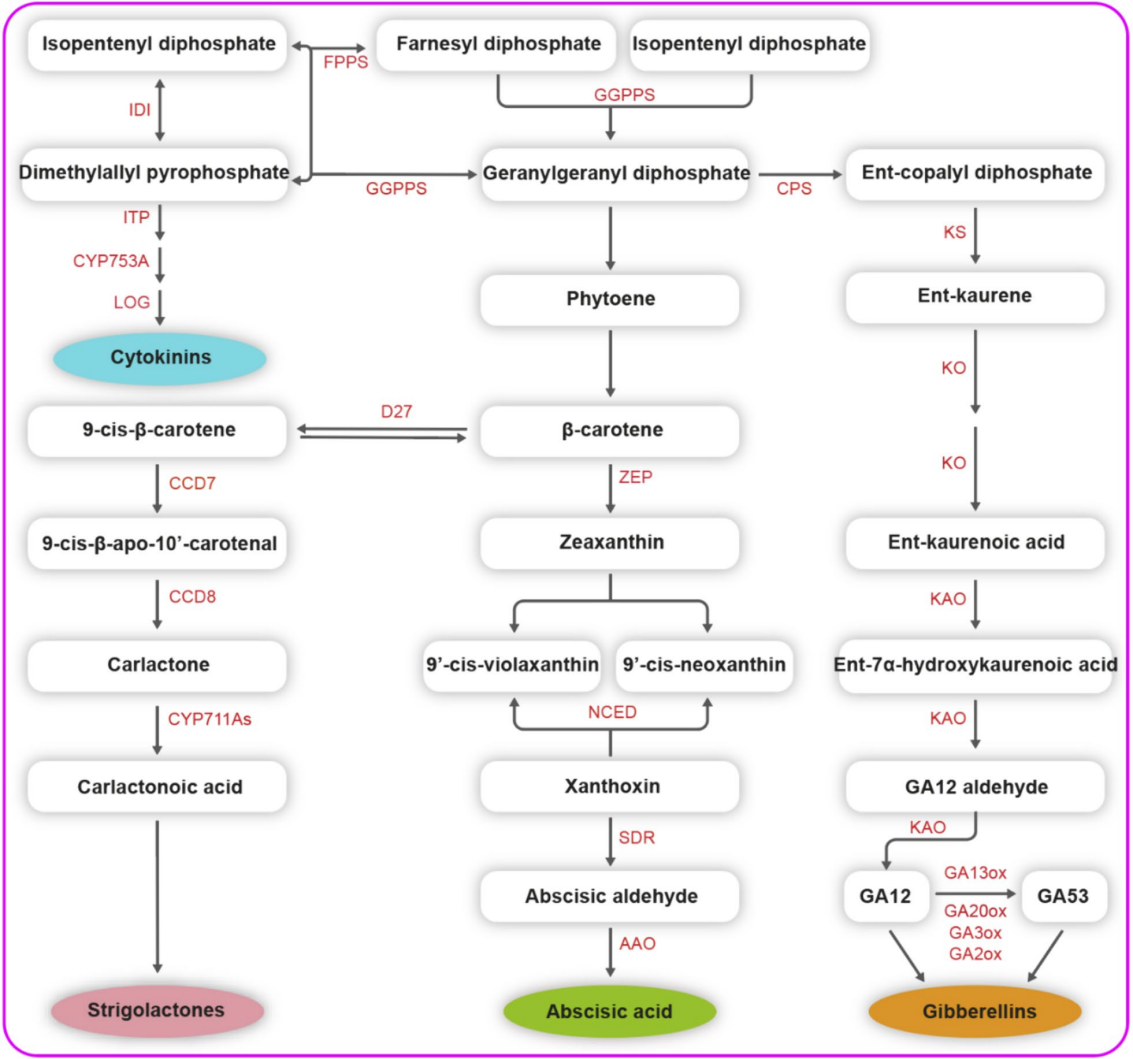
The biosynthetic pathway of CKs, ABA, GAs and SLs in plants. IDI: isopentenyl pyrophosphate isomerase, IPT: isopentenyl transferase, LOG: Lonely Guy, FPPS: farnesyl diphosphate synthase, GGPPS: geranylgeranyl diphosphate synthase, D27: isomerase DWARF27, CCD7: carotenoid cleavage dioxygenase7, CCD8: carotenoid cleavage dioxygenase8, ZEP: zeaxanthin epoxidase, NCED: 9-*cis*-epoxycarotenoid dioxygenase, SDR: short chain dehydrogenase/reductase, AAO: Arabidopsis aldehyde oxidase, CPS: copalyl diphosphate synthase, KS: *ent*-kaurene synthase, KO: *ent*-kaureneoxidase, KAO: *ent*-kaurenoic acid hydroxylase, GA13ox: GA 13-oxidase, GA20ox: GA 20-oxidase, GA3ox: GA 3-oxidase, GA2ox: GA 2-oxidase

CKs are a group of N(6)-substituted adenine derivatives that are widely found in various plants, including *trans*- and *cis*-zeatin, isopentenyl adenine (iP), and dihydro-zeatin (DZ) [110, 111]. Synthetic compounds like 6-benzylaminopurine (BAP), thidiazuron (TDZ), kinetin (Kin), and others exhibit activities similar to those of CKs. CK biosynthesis occurs in different plant organs, including the roots, shoots, and small leaves, particularly in rapidly dividing cells. There are two main pathways for CK biosynthesis: the cytosolic MVA or transfer ribonucleic acid (tRNA) degradation pathway. The former mainly involves three branches, including *cis*-zeatin CKs, and the de novo pathway or the plastidal methylerythritol phosphate (MEP) pathway, which predominantly involves the formation of iP- and *trans*-zeatin-type CKs [111]. Key enzymes in the CK biosynthesis pathway include isopentenyl transferase (IPT), cytochrome P450 enzyme CYP735A, and lonely guy (LOG) [112] (Fig. 5). In *Arabidopsis*, AHK2, AHK3, and CRE1/AHK4 are membrane-localized histidine kinase receptors that detect CK signals. His-Asp phosphorelay (AHP) then transduces the CK signals to activate the transcription factors (RRs) [113] (Fig. 1).

### Gibberellins

GAs are produced in plastids through the MEP pathway from geranylgeranyl diphosphate (GGPP). GGPP is primarily formed from pyruvate and glyceraldehyde 3-phosphate (G3P) (Fig. 5). GGPP formation also requires crosstalk between the MEP and MVA pathways in the cytoplasm during diterpene biosynthesis [114]. GAs are produced in various plant parts, including the root and stem apical meristems, developing leaves, and seed embryos. GA_1_, GA_3_, GA_4_, and GA_7_ are the primary bioactive GAs. GA_4_ and GA_7_, occur naturally, GA_3_ is synthetic, and GA_1_ can be synthesized in plants and fungi [115]. The major elements of GA signaling in *Arabidopsis* consist of the gibberellin-insensitive dwarf1 (GID1)—GA receptor, the DELLA proteins (DELLAs)—growth repressor, and the F-box protein SLEEPY1 (SLY1) [116] (Fig. 1). DELLAs are target elements of the signaling system and are considered to combine signals from different hormones.

### Abscisic acid

In plants, the carotenoid pathway, which is initiated by β-carotene, is mostly used to generate ABA in mature leaves and roots (Fig. 5). β-Carotene precursors, such as isopentenyl pyrophosphate (IPP, also known as isopentenyl diphosphate), farnesyl diphosphate (FPP), and GGPP, are also precursors of the plant hormones CKs, BRs, GAs, and SLs, respectively [117]. The (*R*)-ABA enantiomer, which is unnatural, differs from natural (*S*)-ABA only in the stereochemistry at C-1′ [118]. It is commonly acknowledged that phosphatase clade-A type-2C protein phosphatase (PP2C) is deactivated when ABA connects with pyrabactin-resistant/pyrabactin-resistant-like/regulatory components of abscisic acid receptor (PYR/PYL/RCAR) proteins. Then, sucrose non-fermenting-1 (SNF1)-related protein kinase 2 (SnRK2) begins working [119] (Fig. 1).

### Ethylene

ET, a naturally occurring gaseous hormone, is synthesized from methionine (Met) via *S*-adenosylmethionine (SAM) by ACC synthase (ACS) and ACC oxidase (ACO) in almost all the tissues of higher plants [120]. Ethephon (Eth) was the first synthetic compound to release ET and 1-methylcyclopropen acts as an ET scavenger [2]. The main elements of the ET signaling cascade consist of ET receptors (ETR1, ERS1, ETR2, EIN4, and ERS2) and protein kinases CTR1 and EIN2, which give the transcription factors EIN3, EIL1, and EIL2 a signal (Fig. 1). These proteins then interact with other transcription factors, in particular the ERFs, to trigger ET responses [121].

### Jasmonates

**Fig. 6:**
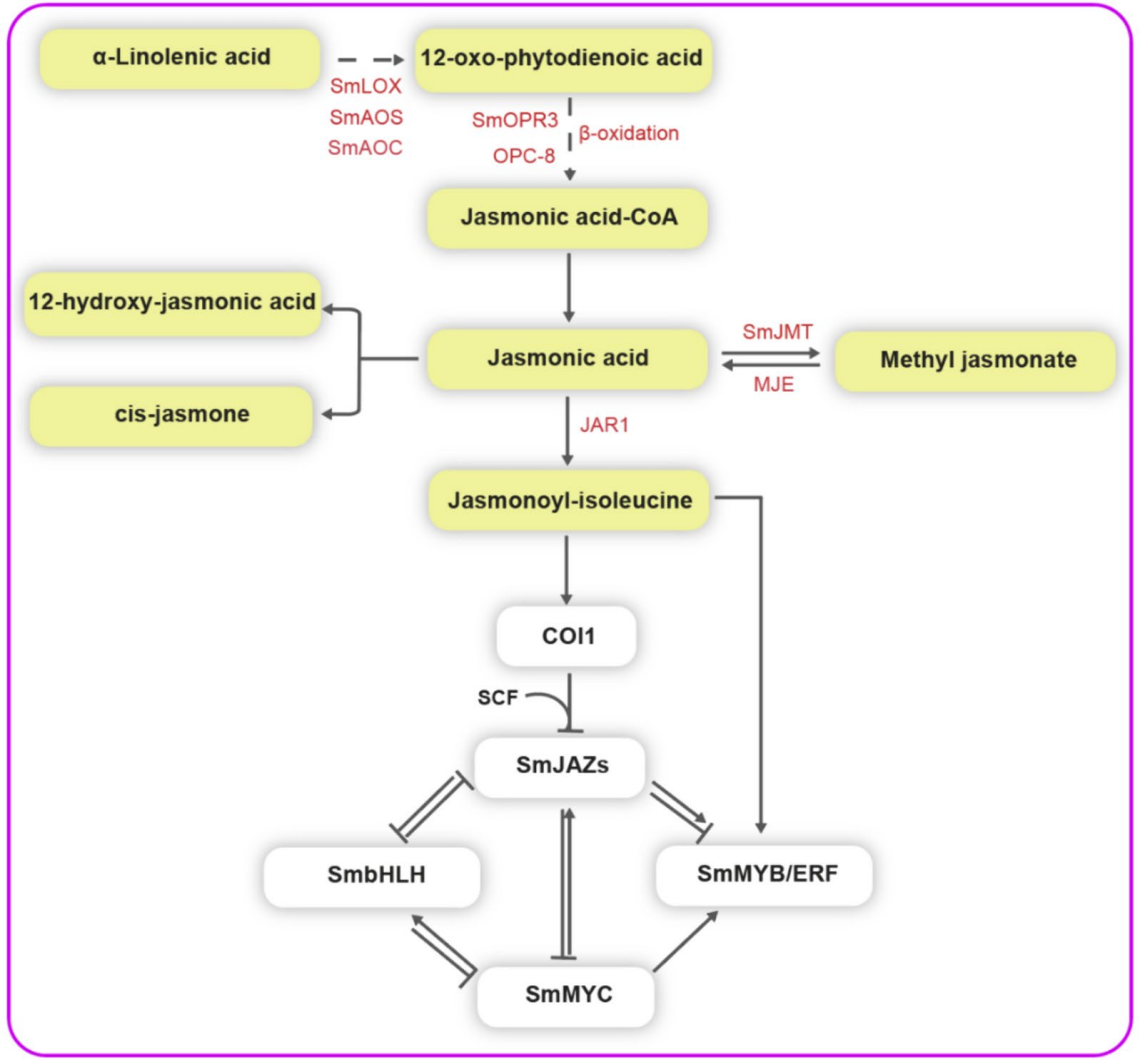
The jasmonic acid biosynthesis and signaling in *S. miltiorrhiza*. SmLOX: lipoxygenase, SmAOS: allene oxide synthase, SmAOC: alleneoxide cyclase, SmOPR3: 12-oxo-phytodienoic acid reductase 3, OPC-8: 3-oxo-2-(2-(Z)-pentenyl)-cyclopentane-1-octanoic, SmJMT: jasmonic acid carboxyl methyltransferase, MJE: methyl jasmonate esterase, JAR1: jasmonic acid-amido synthetase 1, COI1: coronatine insensitive1, SCF: Skp-Cullin-F-box. SmLOX-, SmAOS-, SmAOC-, SmOPR3- and SmJMT-enzyme-coding genes have been identified in *S. miltiorrhiza*. Yellow background box represents the biosynthesis pathway

JA is widely distributed in a variety of higher plants at very low concentrations and is a prototypical member of a set of oxylipid phytohormone that is famous as jasmonates (JAs), which are produced from polyunsaturated fatty acids, mainly α-linolenic acid (α-LeA) [122]. Briefly, lipoxygenase (LOX), allene oxide synthase (AOS), and allene oxide cyclase (AOC) convert α-LeA to 12-oxo-phytodienoic acid (12-OPDA). This is then reduced by 12-oxo-phytodienoic acid reductase 3 (OPR3) and several cycles of β-oxidation to JA [123] (Fig. 6). JA can be catalyzed by jasmonic acid carboxyl methyltransferase (JMT) to form a methyl ester (methyl jasmonate, MeJA) [124], and can also be metabolized to jasmonoyl-isoleucine (JA-Ile) and other compounds [122] (Fig. 6). In leaves and roots, JA biosynthesis is easily activated by insect feeding, pathogen infection, or mechanical damage. The F-box protein coronatine insensitive 1 (COI1)—receptor, the jasmonate ZIM-domain (JAZ)—repressor proteins, and the JAZ-repressed bHLH-type regulators MYC2/3/4 are the elements of the JA signaling pathway [123] (Figs. 1, 6).

### Salicylic acid

SA, originally derived from the leaves and bark of willow trees, is a phenolic phytohormone that can be produced via the isochorismate synthase (ICS) pathway and PAL pathway, both of which require chorismate as a precursor [29, 125]. It contains active analogs, including SA, acetyl SA, methyl SA, and dihydroxy benzoic acid [126]. SA signaling is not a simple linear pathway. Currently, the exact mechanism underlying SA signaling is still poorly understood [127]. *Non-expressors of PR* genes (NPRs), including as *NPR1*, *NPR3*, and *NPR4*, which function as authentic SA receptors, are the most well-characterized SA-binding proteins and directly interact with the TGA transcription factors [128, 129] (Fig. 1).

### Strigolactones and karrikins

SLs and KARs are butenolides. SL biosynthesis primarily synthetize in the roots and is initiated in the plastids by the isomerase DWARF27 (D27). This enzyme converts all *trans*-β-carotene to 9-*cis*-β-carotene. Sequentially, carotenoid cleavage dioxygenase 7 (CCD7) and CCD8 act on this substrate to produce carlactone (CL). CL is catalyzed by the CYP711A subfamily of cytochrome P450 (CYP450) enzymes, eventually producing a different series of SLs [130] (Fig. 5). GR24 is a synthetic analog of SLs that demonstrates higher potency and stability compared with natural SLs. It is widely used and serves as a standard and model compound for studying the characteristics and functions of SLs in plant physiology due to its accessibility and universality [131, 132]. SL signaling is mainly mediated by DWARF14 (D14), MAX2, and the Skp1-Cullin-F-box (SCF) E3 ubiquitin ligase complex, which degrades the transcriptional repressors SMXL6/7/8 (suppressor of max2 1 (smax1)-like 6/7/8) in *Arabidopsis* [32] (Fig. 1). KARs impact various facets of plant growth and development. They are originally identified to be seed germination stimulators in post-fire species after being released from burning plant debris [133]. There might be similarities between the processes of SL and KAR signaling. KAI2, a paralog of SL receptor D14, is widely recognized as a receptor for exogenous KARs and unidentified endogenous KLs. It binds to the F-box protein MAX2 to target SMAX1 and SMXL2 in *Arabidopsis* [134].

### Nitric oxide

NO is a crucial compound for gas signaling. Sodium nitroprusside (SNP) is often regarded as a NO donor and has been used to investigate its diverse biological regulatory functions [135]. In plants, NO biosynthesis is a complicated process and can occur via a variety of oxidative and reductive pathways [3, 39]. However, it is unclear what metabolic processes underlie variations in NO levels in plants. NO signaling appears to be mediated primarily by secondary messengers, including cyclic guanosine monophosphate (cGMP), cyclic ADP-ribose (cADPR), and Ca^2+^ mobilization [9] (Fig. 1); however, the specific mechanism is not clear.

### Polyamines

PAs are a class of aliphatic nitrogenous bases with low molecular weight that are present in nearly every live cell, such as putrescine (Put), spermidine (Spd), and spermine (Spm) that are the most prevalent compounds [136]. Almost all free-living eukaryotes synthesize Put directly from ornithine, which is then catalyzed by spermidine synthase and spermine synthase to form Spd and Spm. They possess an additional biosynthetic pathway for Put formation via the arginine pathway, which involves arginine decarboxylase, agmatine iminohydrolase and *N*-carbamoylputrescine amidohydrolase [137]. PA signaling is associated with the direct interaction of various metabolic processes and complex hormonal crosstalk, integrated with the processes of reactive oxygen species (ROS) signaling, NO production, the regulation of ion channel activity, and Ca^2+^ homeostasis [138].

## Effects of PGRs on active compound production in *S. miltiorrhiza*

It is highly interesting to explore the active pharmacological compounds in medicinal plants. PGRs have a major impact on how medicinal plants produce secondary metabolites [139]. Significant reduction of platycogenin-type saponins has been found in platycodon roots treated with paclobutrazol [140]. In *S. miltiorrhiza*, some PGRs also regulate the active compound production (Table 2). For example, 10 μΜ of IAA considerably enhanced the formation of lateral roots and the production of cryptotanshinone, tanshinone I, and tanshinone IIA [141]. TDZ (0.1 or 0.5 mg/L) has demonstrated a notable improvement in the survival rate of *S. miltiorrhiza* regenerated plantlets. The extracts of the roots of these regenerated plantlets contained salvianolic acid B, dihydrotanshinone I, cryptotanshinone, tanshinone I, and tanshinone IIA [142]. After treatment with N^6^-benzyladenine (0.2 mg/L, the same chemical as BAP), the level of cryptotanshinone in *S. miltiorrhiza* calli reached the maximum [143]. Treatment with 50 mg/L of GA has been found to significantly increase the dihydrotanshinone I, tanshinone I, and tanshinone IIA contents. In contrast, treatment with 100 μΜ of GA_3_ significantly improved the content of salvianolic acid B, rosmarinic acid, dihydrotanshinone I, cryptotanshinone, tanshinone I, and tanshinone IIA in hairy roots of *S. miltiorrhiza* [144, 145]. A previous study has indicated that 10–150 μΜ of GA_3_ only enhanced the level of salvianolic acid B and rosmarinic acid in hairy roots of *S. miltiorrhiza* [146]. The reasons for these differences are worth investigating further. Moreover, 50–200 μΜ of ABA promoted the production of salvianolic acid A, salvianolic acid B, rosmarinic acid, caffeic acid, dihydrotanshinone I, cryptotanshinone, tanshinone I, and tanshinone IIA in *S. miltiorrhiza* hairy roots [147–150]. Additionally, it induced the transcription of numerous differentially expressed genes (DEGs) associated with ABA biosynthesis and signal transduction [151]. It appears that low concentrations of ABA are more likely to regulate the accumulation of phenolic acids, whereas high concentrations of ABA are more likely to regulate the accumulation of tanshinones. However, the regulatory mechanism is largely unknown. By applying 0.05–0.50 mM of ET to *S. miltiorrhiza* calli for 60 days and 200 μg/L of ET to hairy roots for eight days, a notable rise in the quantity of dihydrotanshinone I, cryptotanshinone, tanshinone I, and tanshinone IIA has been found [144, 152], and 170 *AP2/ERF* genes have been identified [153]. In *S. miltiorrhiza* hairy roots, treatment with 50 or 100 μM of ET for 6 days has also positively regulated the level of salvianolic acid B, rosmarinic acid, and caffeic acid [146]. A recent report revealed that adding 70 μM of ET to *S. miltiorrhiza* hairy roots for 50 days inhibited the amount of dihydrotanshinone, cryptotanshinone, and tanshinone I [154]. The various effects of ET on the active compounds could mainly be attributed to differences in the plant culture system, concentration, and duration of ET.

**Table 2:** The effect of PGRs on the active compounds of *S. miltiorrhiza*

PGRs	Concentration	Culture system	Treatment time	Phenolic acids content	Tanshinones content	References
IAA	10 μΜ	Seedlings in pots	42 days		Cryptotanshinone↑	[141]
					Tanshinone I↑	
					Tanshinone IIA↑	
TDZ	0.1 or 0.5 mg/L	Regenerated plants	6 weeks	Salvianolic acid B, 3064.5 μg/g DW	Tanshinone IIA, 357.5 μg/g DW	[142]
					Dihydrotanshinone I, 149.5 μg/g DW	
					Cryptotanshinone, 249.5 μg/g DW	
					Tanshinone I, 125 μg/g DW	
BAP	0.2 mg/L	Callus	60 days		Cryptotanshinone ↑	[143]
GA	50 mg/L	Hairy roots	8 days		Dihydrotanshinone↑	[144]
					Tanshinone I↑	
					Tanshinone IIA↑	
GA_3_	100 μΜ	Hairy roots	6 days	Rosmarinic acid↑	Dihydrotanshinone↑	[145]
				Salvianolic acid B↑	Tanshinone I↑	
					Tanshinone IIA↑	
					Cryptotanshinone↑	
GA_3_	10, 50, 100, 150 µM	Hairy roots	6 days	Rosmarinic acid↑		[146]
Eth	200 μg/L	Hairy roots	8 days		Dihydrotanshinone↑	[147]
					Tanshinone I↑	
Eth	50 and 100 μM	Hairy roots	6 days	Caffeic acid↑		[146]
				Rosmarinic acid↑		
				Salvianolic acid B↑		
Eth	0.05–0.50 mM	Callus	60 days		Dihydrotanshinone↑	[152]
					Cryptotanshinone↑	
					Tanshinone IIA↑	
Eth	70 μM	hairy roots	50 days		Dihydrotanshinone↓	[154]
					Tanshinone I↓	
					Cryptotanshinone ↓	
ABA	50 μM	Hairy roots	6 days	Caffeic acid↑		[146]
				Rosmarinic acid↑		
				Salvianolic acid B↑		
ABA	80 μΜ	Hairy roots	16 days	Salvianolic acid B↑		[147]
				Salvianolic acid A↑		
ABA	200 μΜ	Hairy roots	6 days		Dihydrotanshinone↑	[148, 149]
					Tanshinone I↑	
					Tanshinone IIA↑	
					Cryptotanshinone↑	
ABA	50 μM	Hairy roots	9 days	Rosmarinic acid↑	Dihydrotanshinone↑	[150]
				Salvianolic acid B↑	Cryptotanshinone↑	
					Tanshinone I↑	
					Tanshinone IIA↑	
MeJA	100 μM	Hairy roots	12 days	Rosmarinic acid ↑		[155]
				Lithospermic acid B ↑		
MeJA	100 μM	Hairy roots	6 days		Dihydrotanshinone↑	[156]
					Cryptotanshinone ↑	
					Tanshinone I ↑	
					Tanshinone IIA ↑	
MeJA	100 μM	Hairy roots	9 days	Rosmarinic acid↑		[157]
				Salvianolic acid B↑		
MeJA	100 μM	Hairy roots	9 days		Cryptotanshinone↑	[158]
					Tanshinone I↑	
					Tanshinone IIA↑	
MeJA	100 μM	Hairy roots	9 days	Rosmarinic acid↑	Tanshinone I↓	[160]
				Salvianolic acid B↑	Tanshinone IIA↓	
					Cryptotanshinone↑	
					Dihydrotanshinone↑	
MeJA	100 μM	Hairy roots	9 days	Rosmarinic acid↑	Dihydrotanshinone↑	[161]
				Salvianolic acid B↑	Cryptotanshinone↑	
				Caffeic acid↑		
MeJA	50–500 μM	Callus	60 days	Caffeic acid↑	Dihydrotanshinone↑	[163]
				Rosmarinic acid↑	Cryptotanshinone ↑	
				Salvianolic acid B↑	Tanshinone I↑	
					tanshinone IIA ↑	
SA	0.16 mM	Cell cultures	168 h	Salvianolic acid B↑		[164]
				Caffeic acid↑		
SA	50 μM	Hairy roots	72 h	Rosmarinic acid↑		[165]
				Salvianolic acid B↑		
SA	0.16 mM	Cell cultures	48 h	Rosmarinic acid↑		[166]
				Salvianolic acid B↑		
				Caffeic acid↑		
SA	100 μM	Hairy roots	144 h		Dihydrotanshinone↑	[168]
					Cryptotanshinone ↑	
					Tanshinone I ↑	
					Tanshinone IIA ↑	
SA	0.4 mM	Callus	80 days		Dihydrotanshinone↑	[169]
					Cryptotanshinone ↑	
					Tanshinone I ↑	
					Tanshinone IIA ↑	
SA	22.5 mg/L	Cell cultures	6 days	Rosmarinic acid↑		[192]
SA	22 mg/L	Cell cultures	8 days	Salvianolic acid B↑		[193]
NO	100 μM	Hairy roots	6 days		Dihydrotanshinone↑	[156, 171]
					Cryptotanshinone ↑	
					Tanshinone I ↑	
					Tanshinone IIA ↑	
Put	10, 50, 100 mg/L	Hairy roots	12 days	Salvianolic acid A↑		[147]
Spd				Salvianolic acid B↑		
Spm						
SL	100 μM	Seedling-AMF symbiont	210 days		Tanshinone IIA↑	[170]
					Cryptotanshinone↑	
ABA + GA_3_	50 μM + 100 μM	Hairy roots	6 days	Rosmarinic acid↓		[146]
				Salvianolic acid B↑		
				Caffeic acid↑		
ABA + ET	50 μM + 50 μM			Caffeic acid↑		
				Rosmarinic acid↑		
				Salvianolic acid B↑		
ET + GA_3_	50 μM + 100 μM			Rosmarinic acid↑		
				Salvianolic acid B↑		

Different MeJA treatments have been demonstrated to greatly raise the level of phenolic acids and/or tanshinones in the hairy roots and calli of *S. miltiorrhiza*. The synthesis of phenolic acids in hairy roots of *S. miltiorrhiza* is more efficiently improved than that of tanshinones [155–163]. Compared with other PGRs, the effects and regulatory mechanisms of JAs on the active compound production of *S. miltiorrhiza* have been extensively studied. SA has been reported to induce the synthesis of salvianolic acid B, rosmarinic acid, and caffeic acid in *S. miltiorrhiza* hairy roots and cell cultures in concentration- and time-dependent manners [164–166]. The accumulation of these compounds leads to the induction of many DEGs involved in SA signaling, antioxidant systems, hormone biosynthesis and signaling, defense-related CYP450, and ATP-binding cassette (ABC) [167]. In addition, SA has been reported to increase the level of dihydrotanshinone I, cryptotanshinone, tanshinone I, and tanshinone IIA; however, their yields were relatively low [168, 169]. To date, only one report of SL in *S. miltiorrhiza* has been published. The study revealed that under arbuscular mycorrhizae and GR24 treatment, the maximum amount of tanshinone IIA and cryptotanshinone production was shown in (NH_4_)_2_SO_4_-treated *S. miltiorrhiza* roots [170]. SNP significantly stimulated tanshinone production and increased cryptotanshinone and tanshinone IIA accumulation [156]. It is possible that NO signaling is crucial to the production of dihydrotanshinone I, cryptotanshinone, tanshinone I, and tanshinone IIA triggered by water deficiency in *S. miltiorrhiza* hairy roots, mainly through stimulation of the MEP pathway [171]. Both ABA and PAs promote salvianolic acid synthesis in the *S. miltiorrhiza* hairy roots and that Put and Spd are superior to Spm in promoting salvianolic acid B and salvianic acid A synthesis [147]. These studies suggest that PGRs effectively enhance the accumulation of salvianolic acids and/or tanshinones in *S. miltiorrhiza*. Moreover, the yield of salvianolic acids and/or tanshinones is influenced by the type, dosage, and application time of the PGRs, as well as the plant cultivation system. However, the specific mechanisms involved require further investigation.

## Mechanisms by which PGRs regulate active compound production in *S. miltiorrhiza*

According to the previous studies, PGRs regulate the physiological, biochemical, and transcriptional levels of active compounds in *S. miltiorrhiza*. Furthmore, PGRs play a vital role in the active compound production of *S. miltiorrhiza* through transcription factors, enzymes, enzyme-encoding genes, signal transduction, post-transcriptional regulation, and crosstalk.

### PGRs regulate active compound production in *S. miltiorrhiza* through transcription factors

**Fig. 7:**
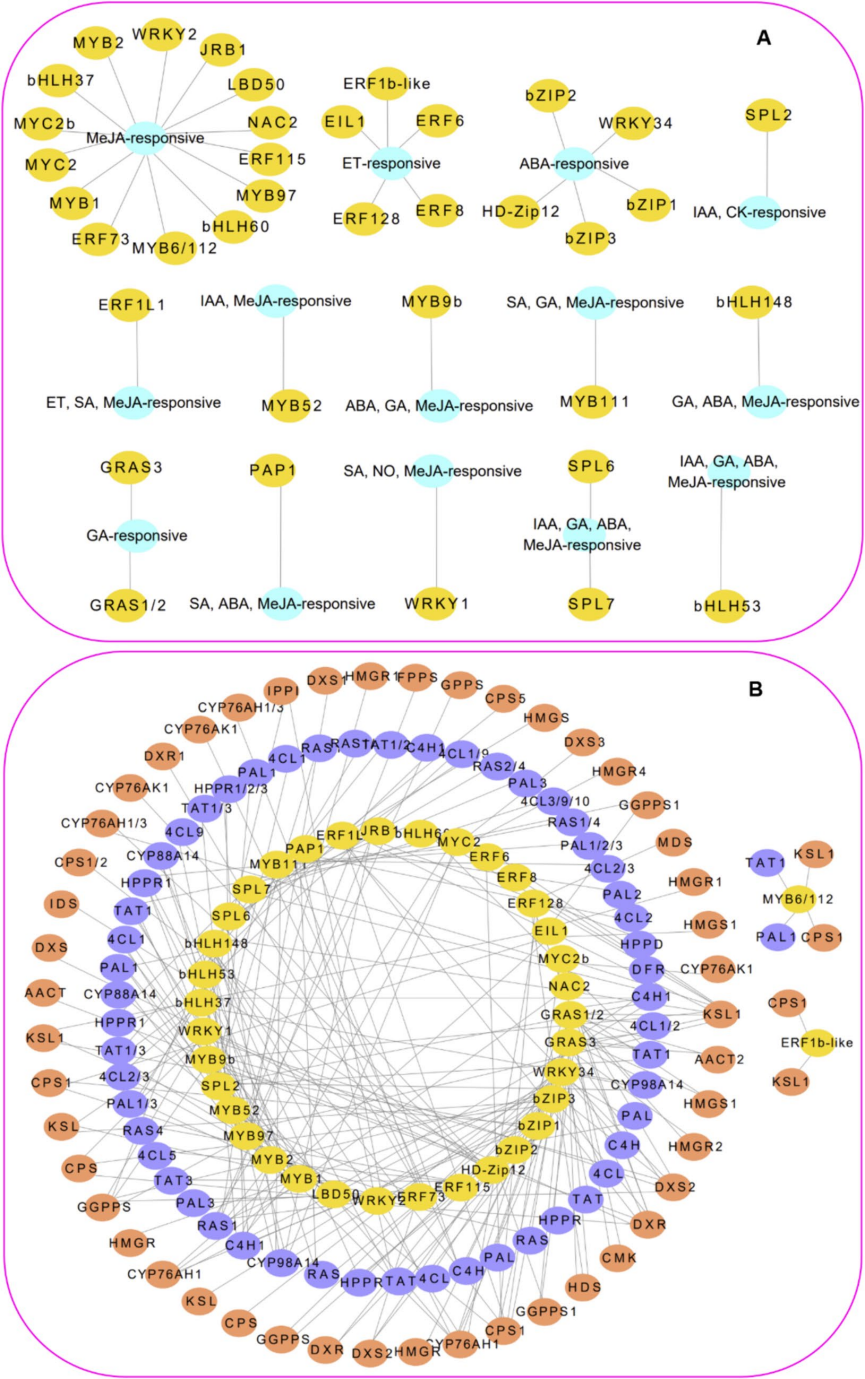
The PGRs-responsive transcription factors and related genes in *S. miltiorrhiza*. Light blue circle represents the PGRs. Yellow circle represents the transcription factors. Blue circle represents phenolic acid biosynthesis genes. Red circle represents tanshinone biosynthesis genes. The networks were visualized using Cytoscape software (v. 3.10.2) (https://cytoscape.org/)

Considerable advancements have been achieved in studying the roles and regulatory mechanisms of PGRs in the secondary metabolites of *S. miltiorrhiza* associated with transcription factors (Fig. 7). For example, GA-responsive SmGRAS1/2 plays an inhibitory role in root growth and the biosynthesis of rosmarinic acid and salvianolic acid B but it promotes the production of cryptotanshinone, dihydrotanshinone I, tanshinone I, and tanshinone IIA by binding to the *SmKSL1* promoter in *S. miltiorrhiza* hairy roots [172]. In contrast, SmGRAS3 promotes the biosynthesis of cryptotanshinone, dihydrotanshinone I, and tanshinone I [173]. Some transcription factors that respond to ABA also have an important effect on the active compound production of *S. miltiorrhiza*. For instance, the SmWRKY34-SmbZIP3 negatively regulates the production of rosmarinic acid, salvianolic acid B, cryptotanshinone, dihydrotanshinone I, tanshinone I, and tanshinone IIA by interacting with *SmTAT* and two transcription factors, SmERF128 and SmMYB9b that associated with tanshinone biosynthesis [174]. SmbZIP1 positively promotes the biosynthesis of rosmarinic acid and salvianolic acid B by enhancing the expression of biosynthetic genes, such as *SmC4H1*, while decreasing the level of cryptotanshinone, dihydrotanshinone, tanshinone I, and tanshinone IIA, mainly by suppressing the expression of biosynthetic genes, such as *SmGGPPS* [175]. SmbZIP2 reduced the amount of rosmarinic acid and salvianolic acid B by linking to the *SmPAL* promoter and physically interacts with several SnRK2s, including SnRK2.3, SnRK2.4, SnRK2.6, and SnRK2.10 [176]. SmHD-Zip12 positively regulates the level of cryptotanshinone, dihydrotanshinone I, tanshinone I, and tanshinone IIA by altering the transcription of some key enzyme-coding genes, such as *SmAACT*, *SmDXS*, *SmGGPPS*, *SmCPS1*, *SmCYP76AH1*, *SmCYP76AH3*, and *SmCYP76AK1* [177].

Some transcription factors responsive to JA, such as the MYB, ERF, WRKY, NAC, GATA, and LBD family members, have also been shown to regulate the synthesis of secondary metabolites in *S. miltiorrhiza*. SmMYB1/2 positively regulates the biosynthesis of rosmarinic acid, caffeic acid, salvianolic acid A, and salvianolic acid B by binding to the promoter of *CYP98A14*. In contrast, by binding to and activating the promoters of *SmPAL1*, *SmTAT1*, *SmCPS1*, and *SmKSL1*, SmMYB97 positively regulates the biosynthesis of rosmarinic acid, salvianolic acid B, cryptotanshinone, dihydrotanshinone, tanshinone I, and tanshinone IIA [178–180]. *SmMYB6*, *SmMYB97*, and *SmMYB112*, which are regulated by Smi-miR858a, can activate the genes linked to the biosynthesis of MeJA, rosmarinic acid, salvianolic acid B, cryptotanshinone, dihydrotanshinone, tanshinone I, and tanshinone IIA [181]. By directly binding to the *SmRAS1* promoter, SmERF115 positively regulates salvianolic acid B biosynthesis [182]. Additionally, by directly linking to the promoters of *SmDXR1*, *SmCPS1*, *SmKSL1*, and *SmCYP76AH3*, SmERF73 induces the biosynthesis of cryptotanshinone, dihydrotanshinone, tanshinone I, and tanshinone IIA. This step is partly controlled by the JA signaling pathway through cooperation with SmJAZ3 in *S. miltiorrhiza* [183]. *SmWRKY2* overexpression has been reported to significantly activate *SmDXS2* and *SmCPS* expression; however, binding only to the *SmCPS1* promoter increase the accumulation of cryptotanshinone, dihydrotanshinone, tanshinone I, and tanshinone IIA in *S. miltiorrhiza* hairy roots [184]. *SmLBD50* overexpression inhibits the production of total phenolics, flavonoids, and anthocyanins in *S. miltiorrhiza*, and SmLBD50 interacts with SmJAZ1, SmMYB36/97, SmbHLH37, and SmMYC2a/b [185]. SmNAC2 inhibits tanshinone biosynthesis in *S. miltiorrhiza* [186], whereas SmGATA08, SmGATA09, and SmGATA13 may be crucial for tanshinones and phenolic acids biosynthesis [187].

In general, PGRs regulate active compound production in *S. miltiorrhiza* through transcription factors directly binding to the promoters of genes related to the active compound biosynthesis. These genes include key enzyme-encoding genes of the biosynthetic pathway of active compounds, or other transcription factors. However, the effects and mechanisms of these PGRs on the transcription factors need to be further investigated.

### PGRs regulate active compound production in *S. miltiorrhiza* through enzymes or enzyme-encoding genes

In *S. miltiorrhiza*, PGRs also regulate secondary metabolites by modifying the activity of key enzymes or expression of key enzyme-encoding genes of secondary metabolic biosynthetic pathways. For example, exogenous IAA significantly promote the concentration of tanshinone and stimulate the transcription of several key enzyme-encoding genes involved in the tanshinone biosynthesis pathway, such as *SmCPS1*, *SmDXR*, *SmDXS2*, and *SmKSL1* [141]. Both GA_3_ and ET have been found to be effective in promoting phenolic acid accumulation and increasing PAL and TAT activities in the hairy roots of *S. miltiorrhiza* [146]. GAs and ET treatments also improve the transcription of *SmKSL* and *SmCPS1*, leading to tanshinone accumulation [144, 145, 152]. Most recently, ET was found to decrease the transcription of *SmCPS1*, *SmGGPPS*, and *SmCYP76AH1* as well as the production of tanshinones [150]. MeJA[155–157, 160, 161, 168, 188–191], ABA [146–151, 188, 191], and SA [165, 166, 168, 169, 188–193] have been shown to induce the transcription of some genes linked to phenolic acids and tanshinones biosynthesis, such as *SmPAL*, *SmC4H*, *SmRAS*, *SmTAT*, *SmHMGR*, *SmDXS*, *SmDXR*, *SmCPS*, *SmGGPPs*, and *SmKSL*. In addition, both MeJA and SA regulate the transcription of *4-hydroxyphenylpyruvate dioxygenase* (*Smhppd*), which significantly contributes to the accumulation of rosmarinic acid in *S. miltiorrhiza* [194]. Transcriptional data analysis has suggested that MeJA improves the transcription of some genes linked to tanshinone and phenolic acid biosynthesis, such as the *2C methyl-D-erythritol 2,4-cyclodiphosphate synthase* (*SmMEC* or *SmMDS*) and the *caffeoyl*-*CoA O*-*methyltransferase* (*SmCCoAOMT*) genes. The transcription of these genes had a significant impact on the level of tanshinones and phenolic acids in *S. miltiorrhiza* [163, 195, 196]. NO has been found to promote tanshinone accumulation and enhance *SmHMGR* and *SmDXR* expression [156, 171].

In *S. miltiorrhiza*, some genes related to the PGRs biosynthetic pathway regulate secondary metabolites. For example, in the JAs biosynthetic pathway, overexpression of *SmAOC* positively regulates the biosynthesis of tanshinone IIA, rosmarinic acid, and lithospermic acid B in *S. miltiorrhiza* hairy roots [197]. Overexpression of *SmJMT* increases the level of salvianolic B and rosmarinic acid by simultaneously activating the expression of *SmPAL1*, *SmC4H*, *Sm4CL3*, *SmTAT*, *SmHPPR*, *SmRAS*, and *SmCYP98A* and raising the endogenous levels of MeJA [198]. In *S. miltiorrhiza*, 23 potential JA biosynthesis-related genes have been reported, including nine *SmLOXs*, seven *SmAOSs*, two *SmAOCs*, and five *SmOPR3s* [199]. MeJA has been proven to be effective in the accumulation of salvianolic acids and/or tanshinones in *S. miltiorrhiza*, and one of the *SmAOCs* related to the production of tanshinones and phenolic acids has been previously published [196]; therefore, these JA biosynthetic genes may play vital roles in the accumulation of tanshinones and/or phenolic acids. GA may also affect tanshinones production by changing the transcription of some genes including *ent*-*copalyl diphosphate synthase* (*SmCPS*), *ent*-*kaurene synthase* (*SmKS*) and *ent*-*kaurene oxidase* (*SmKO*), which encode enzymes in the biosynthetic pathway of GA in *S. miltiorrhiza* [200, 201]. Twenty-two candidate genes of GA metabolism pathway have been systematically identified, including one *SmKO*, two *SmKAOs*, six *SmGA20oxs*, two *SmGA3oxs*, and 11 *SmGA2oxs*. These genes play significant roles in GA production and the interaction between GA accumulation and tanshinone production in *S. miltiorrhiza* [114].

In addition, some MeJA-responsive proteins, such as the phenolic acid-related Kelch domain-containing F-box (KFB) protein SmKFB5 [202] and the tanshinone-related protein *Lycopersicon esculentum* (tomato) prosystemin (LePS) [203], have been found to mediate the degradation of SmPAL and increase the expression of *SmDXS1*, *SmDXR*, *SmHMGR1*, *SmCPS1*, *SmKSL1*, *SmCYP76AH1*, *SmCYP71D441*, *SmSDR1*, *SmGGPPS1*, and *Sm2ODD8* in *S. miltiorrhiza*. MeJA-responsive SmLAC3 plays a positive role in salvianolic B biosynthesis and rosmarinic acid production. It also up-regulates the transcription of *SmTAT1*, *Sm4CL1*, and *Sm4CL2* in *S. miltiorrhiza* [204]. The GA-responsive SmGASA4 (gibberellic acid-stimulated Arabidopsis) up-regulates the expression of *Sm4CL1*, *SmC4H1*, *SmCPR1*, *SmHPPR1*, *SmPAL1*, and *SmTATA1*, but inhibits the expression of *SmAACT*, *SmHMGS*, *SmMK*, *SmPMK*, *SmMDS*, *SmDXS*, *SmDXR*, *SmMCT*, and *SmMCS* in *S. miltiorrhiza* [205].

Taken together, PGRs directly modulate the activity of key enzymes or expression of key enzyme-encoding genes of active compound biosynthetic pathways, or indirectly influence the activity of key enzymes or expression of key enzyme-encoding genes by the transcription of PGRs biosynthetic pathway genes or other protein genes, to regulate active compound production in *S. miltiorrhiza*. However, little is known about how these PGRs regulate the enzyme activity and enzyme-encoding gene expression.

### PGRs regulate active compound production in *S. miltiorrhiza* via signal transduction

By influencing the transcription of genes associated with PGR signal transduction, PGRs also regulate secondary metabolites of *S. miltiorrhiza*. For example, exogenous IAA significantly promotes tanshinone content and alters the transcription of AUX biosynthesis- and signal transduction-related genes, including *AUX/IAAs*, *GH3s*, and *SAURs* [141]. Genome-wide investigation has revealed 23 *AUX/IAA* genes and 25 *ARF* genes in *S. miltiorrhiza* [206, 207]. These genes may be important for the growth and development of plants as well as the final content of secondary metabolites in *S. miltiorrhiza*. Moreover, ABA signaling genes have been found to modulate the biosynthesis of phenolic acids and tanshinones. For instance, *SmSnRK2.6*, which is linked to ABA signaling, acts as an inducer in the production of rosmarinic acid and salvianolic acid B through interactions with *SmAREB1* in the hairy roots of *S. miltiorrhiza* [208]. Recently, Ding et al. have reported that SA regulates the level of salvianolic acid B and rosmarinic acid via the SmNPR1-SmTGA2/SmNPR4 or SmNPR4-SmTGA5 modules in *S. miltiorrhiza* hairy roots [209, 210]. SA also has been shown to significantly promote rosmarinic acid accumulation through secondary messengers associated with signal transduction, including H_2_O_2_, NO, and Ca^2+^ [192, 193, 211].

In the process of JA-induced active compound biosynthesis in *S. miltiorrhiza*, the roles of JA signaling components of SmJAZs are redundant, diversified, and pleiotropic [212]. For example, MeJA-responsive *SmJAZ3* and *SmJAZ9* act as repressors of the JA signaling pathway and take part in the tanshinone biosynthesis in hairy roots [213]. SmJAZ3 interacts with SmWD40-170 to reduce the transcription of genes related to the synthesis of rosmarinic acid, salvianolic acid B, cryptotanshinone, and tanshinone IIA. These genes include *SmTAT*, *SmHPPR*, *SmPAL*, *SmC4H*, *Sm4CL*, *SmRAS*, *SmCYP98A14*, *SmDXS*, *SmHMGR*, *SmFPPS*, *SmGGPPS*, *SmCPS*, *SmKSL*, and *SmCYP76AH1* [214]. The SmJAZ9-SmMYB76 complex also modulates the JA-mediated accumulation of caffeic acid, rosmarinic acid, and salvianolic acid B by directly down-regulating the transcription of *SmPAL1*, *Sm4CL2*, and *SmRAS1* [215]. The SmJAZ4-SmMYC2/SmMYB111 module directly interacts with *SmTAT1* and *SmCYP98A14* to regulate salvianolic acid B biosynthesis through JA signaling [216]. In JA-induced the production of rosmarinic acid, salvianolic acid B, cryptotanshinone, dihydrotanshinone I, tanshinone I, and tanshinone IIA, MeJA-responsive SmJAZ8 acts as a negative feedback loop controller and may directly interact with SmMYC2a [217, 218]. Furthermore, the JA signaling bHLH family members also influence the secondary metabolite accumulation in *S. miltiorrhiza* (Fig. 7)*.* For example, JA-responsive SmJRB1 improve the accumulation of salvianolic acid A, salvianolic acid B, and rosmarinic acid by activating *RAS1* [219]. MeJA-responsive SmbHLH37 links to the *SmTAT1* and *SmPAL1* promoters to suppress the salvianolic acid B biosynthesis pathway. This suppression reduces JA signaling and acts adversely with SmMYC2 [220]. MeJA-responsive SmbHLH60 and SmMYC2 form a heterodimer that antagonizes the biosynthesis of rosmarinic acid, salvianolic acid B, caffeic acid, and anthocyanin by inhibiting the expression of *SmTAT1* and *SmDFR* [221]. SmMYC2 positively regulates the biosynthesis of rosmarinic acid, salvianolic acid A, and salvianolic acid B and up-regulates the expression of *SmTAT*, *SmPAL*, *SmHPPR*, *SmC4H*, and *SmRAS* [180, 222], whereas SmMYC2a/2b improves the production of cryptotanshinone, dihydrotanshinone I, tanshinone I, and tanshinone IIA and induces the transcription of *SmCPS1*, *SmKSL1*, *SmCYP76AH1*, *SmCYP76AH3*, and *SmCYP76AK1* [88] (Fig. 7).

Several ERFs associated with ET signaling pathway, have been proven to modulate the level of phenolic acids and tanshinones (Fig. 7). ET-sensitive SmERF6/8/128/SmERF1b-like proteins can enhance the biosynthesis of cryptotanshinone, dihydrotanshinone I, tanshinone I, and tanshinone IIA by regulating the gene expression of *SmCPS1*, *SmKSL1*, and *SmCYP76AH1* from MEP pathway [223–226]. Conversely, overexpression of the ET-responsive transcription factor *EIN3-like 1* (*SmEIL1*) suppresses tanshinone accumulation by inhibiting the transcription of the *SmCPS1* [153]. Furthermore, some genes linked to signal transduction and the level of tanshinones and phenolic acids in *S. miltiorrhiza*, also respond to PGRs. For example, *SmMAPK3* regulates the level of rosmarinic acid and salvianolic acid B via the *SmMAPKK2/4/5/7*-*SmMAPK3*-SmJAZ cascade, in response to SA and MeJA [227]. The expression of *SmGH3.2*, *SmGH3.6*, and *SmGH3.10*, which are involved in the AUX response, is up-regulated by MeJA in *S. miltiorrhiza* [228]. Several *U-box E3* (*UBE3*) genes may be linked with the accumulation of phenolic acids or tanshinones via the ABA signaling pathway [229]. However, to fully understand the regulatory mechanisms of these genes, further studies are needed.

So far, for this regard, the signal transduction of JAs, SA, ABA, and NO that regulate active compound production in *S. miltiorrhiza* is relatively clear. The regulatory mechanism of other PGRs including SLs, KLs, BR, PAs in active compound production in *S. miltiorrhiza*, is still little known. In our opinion, to gain a lot of knowledge of the regulatory mechanism of PGRs in active compound production in *S. miltiorrhiza* via signal transduction, identification of the biosynthesis and signaling transduction pathways of PGRs in *S. miltiorrhiza* should be firstly paid close attention.

### PGRs regulate active compound production in *S. miltiorrhiza* via post-transcriptional regulation

MicroRNAs (miRNAs) act as important regulators of plant development, secondary metabolism, and environmental stimuli through cleavage or translational inhibition [230, 231]. By targeting genes that encode enzymes and transcription factors associated with biosynthetic pathways, miRNAs influence a variety of bioactive compound production, including terpenoids, alkaloids, flavonoids, and phenolic acids [232]. It has been discovered that Mdm-miR858 targets *MdMYB9* and *MdMYBPA1* to take part in the production of anthocyanins in red-fleshed apple plants [233]. miR828 and miR858 have been found to target *VvMYB114* to promote the production of anthocyanin and flavonols in grapes [234]. Many miRNAs have been identified in *S. miltiorrhiza* [232], some of which are known to be responsive to PGRs. For example, miR396b, activated by MeJA, ABA, GA, etc., can function as an important upstream regulator of cell growth and tanshinones and salvianolic acids accumulation in *S. miltiorrhiza* [235]. Overexpression of *MIR160a* might negatively regulate tanshinones biosynthesis by increasing IAA accumulation and decreasing SA and JA levels [236]. Smi-miR12112 is responsive to MeJA treatment and potentially affects phenolic acid biosynthesis by targeting *SmPPOs* [230]. However, the specific regulatory effects of PGR-responsive miRNAs on secondary metabolites accumulation in *S. miltiorrhiza* remain unclear. In *S. miltiorrhiza*, the function of a great many miRNAs is unknown and the response of miRNAs to PGRs is also poorly known, therefore, the study on PGRs regulating active compound production in *S. miltiorrhiza* via post-transcriptional regulation will have great research prospects.

### PGRs regulate active compound production in *S. miltiorrhiza* through crosstalk

**Fig. 8:**
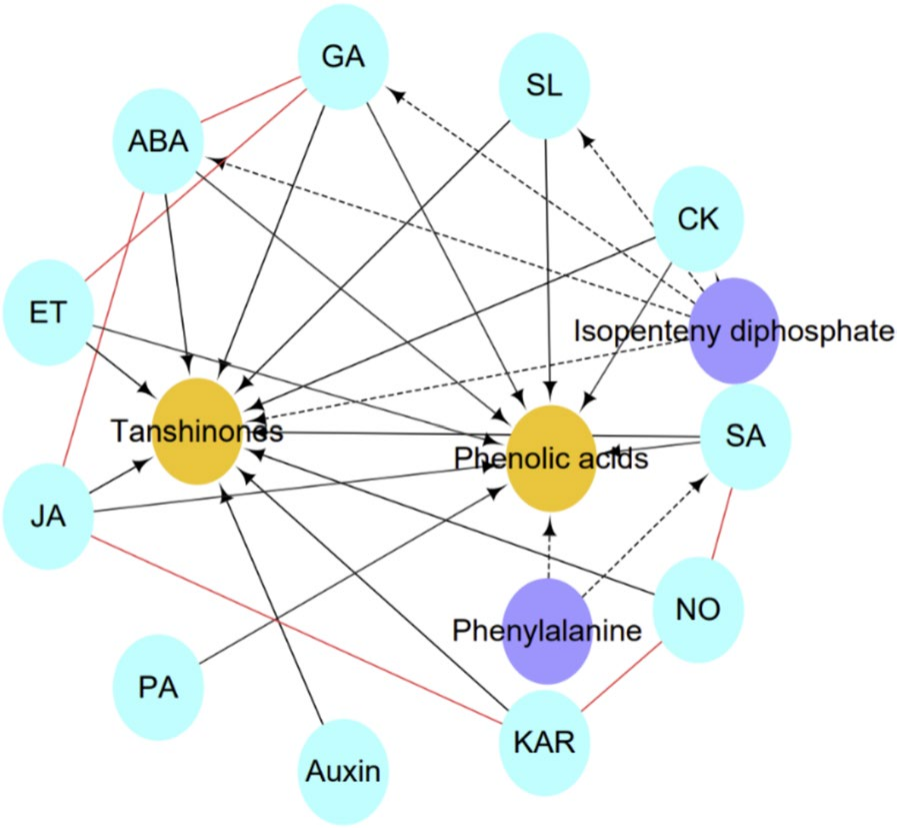
The relationship between PGRs and tanshinones and phenolic acids in *S. miltiorrhiza*. Dashed arrows denote multiple steps in the biosynthetic pathways. Solid arrows represent the regulatory function. Red lines represent the crosstalk of PGRs

Under both normal and stressful circumstances, PGRs are of vital importance in regulating various aspects of plant growth and development and operate within complex networks (Fig. 1). Studies on a variety of plants have suggested the function of PGR crosstalk in the secondary metabolite accumulation. For example, in in vitro shoot cultures of *Bacopa monnieri*, MeJA combined with SA has been demonstrated to considerably raise the biomass and active bacoside A content [237]. Additionally, in the root suspension of *Ajuga bracteosa*, the combined action of MeJA and PAA increases the total phenolic and flavonoid contents [238]. GR24 combined with ABA delays anthocyanin accumulation and reduces the anthocyanin biosynthetic gene expression in grapevine berries [33]. Crosstalk between PGRs has a great impact on active compound accumulation of *S. miltiorrhiza* (Table 2; Fig. 8). For example, endogenous MeJA accumulation is triggered by exogenous application of polyethylene glycol and ABA, which activate the ABA signaling pathway and improve tanshinone synthesis. In contrast, in *S. miltiorrhiza* hairy roots, exogenous MeJA might directly trigger tanshinone synthesis mostly through the MEP pathway [147]. Through a JA-dependent signaling mechanism, NO promotes the synthesis of tanshinone I triggered by KAR [239]. H_2_O_2_ and NO elicited by SA can work alone or together to enhance salvianolic acid B accumulation in SA-stimulated *S. miltiorrhiza* cells [192]. JA participates in the signal transduction pathway that drives the yield of salvianolic acid B and tanshinone in response to KAR stimulation in *S. miltiorrhiza* [239–241]. ABA, GA, and ET are effective in inducing phenolic acid production and increasing PAL and TAT activities in *S. miltiorrhiza* hairy roots, and the synthesis of phenolic compounds driven by ABA and ET requires GA signaling [145].

Moreover, some transcription factors regulate active compound production in *S. miltiorrhiza* by modulating the PGR balance (Fig. 7). For example, SmMYB52 inhibits the expression of key enzyme-encoding genes associated with the IAA biosynthesis and activates key enzyme-encoding genes associated with the JA and salvianolic acid B biosynthesis [242]. Overexpression of *rSmSPL2* has a negative effect on the level of salvianolic acid B and rosmarinic acid in *S. miltiorrhiza* roots. However, it significantly increases the endogenous CK levels and dramatically decreases the endogenous AUX levels by binding to the promoters of *Sm4CL9*, *SmTAT1*, and *SmPAL1* and inhibiting their transcription [243].

In contrast, some transcription factors that influence the active compound production in *S. miltiorrhiza* have been demonstrated to be responsive to at least two types of PGRs (Fig. 7). Overexpression of *SmMYB9b*, activated by ABA, GA, and MeJA, results in a higher content of tanshinones by stimulating the MEP pathway [244]. SmPAP1, the R2R3-MYB transcription factor, is induced by MeJA, SA, and ABA. By connecting with SmMYC2 and turning on the transcription of *SmPAL* and *SmC4H* in the transgenic line roots, it takes part in regulating the biosynthetic pathways of rosmarinic acid, salvianolic acid B, total phenolics, and total flavonoids [245]. SmMYB111 responds to SA, GA, and MeJA and serves to positively regulate the rosmarinic acid and salvianolic acid B biosynthetic pathways by interacting with SmTTG1 and SmbHLH51 [246]. Both SmSPL6 and SmSPL7 respond to treatment with IAA, GA, MeJA, and ABA. The former directly connects with *Sm4CL9* and *SmCYP98A14*, promoting the yield of rosmarinic acid and salvianolic acid B, whereas the latter binds to the promoters of *Sm4CL9* and *SmTAT1*, inhibiting salvianolic acid B biosynthesis [247, 248]. ABA induces SmbHLH148 at high levels, whereas MeJA and GA induce it at a moderate level. By inducing the transcription of key enzyme-encoding genes linked to the active compound biosynthesis, such as *SmHPPR*, *SmRAS*, *SmCYP98A14*, *SmGGPPS*, *SmCPS1*, *SmCPS5*, *SmKSL1*, and *SmCYP76AH1*, its overexpression improves the level of dihydrotanshinone I, cryptotanshinone, tanshinone I, caffeic acid, rosmarinic acid, and salvianolic acid B in hairy roots of *S. miltiorrhiza* [249]. SmbHLH53 is responsive to IAA, ABA, GA_3_, and MeJA. It forms a homodimer and heterodimer with SmbHLH37. By binding to the *SmTAT1/PAL1/4CL9* promoter, it inhibits JA signaling, antagonizes SmMYC2, and regulates the content of salvianolic acid B [219, 250]. SmERF1L1 is activated by MeJA, yeast extract, SA, and ET. This activation increases the dihydrotanshinone and cryptotanshinone contents and decreases the rosmarinic acid and salvianolic acid A contents by binding to the promoter of *SmDXR* in *S. miltiorrhiza* [251]. SmWRKY1 is responsive to SA, MeJA, and NO; participates in the regulation of cryptotanshinone, dihydrotanshinone I, tanshinone I, and tanshinone IIA biosynthesis; and acts as an inducer by activating *SmDXR* in the MEP pathway [252]. SmWRKY42-like proteins respond significantly to exogenous GA and Eth treatment. It may be crucial in enhancing the medicinal properties of *S. miltiorrhiza* [253]. SmGRAS1-5, which may contribute to the tanshinone accumulation, is regulated by GA and ET signaling in *S. miltiorrhiza* [143].

Furthermore, some enzyme-encoding genes regulating the level of phenolic acids and tanshinones have been shown to be responsive to more than one type of PGR. *SmDXR*, an enzyme that is essential for the tanshinone biosynthetic pathway, responds to MeJA and SA [254]. SmCYP76AK2 and SmCYP76AK3 could respond to MeJA, SA, which significantly reduces the content of cryptotanshinone, tanshinone IIB, dihydrotanshinone, tanshinone I, and tanshinone IIA in *S. miltiorrhiza* mutants [255]. SmLAC25 is responsive to MeJA, AUX, ABA, and GA stimuli, and its overexpression promotes lignin accumulation and decreases rosmarinic acid and salvianolic acid B concentrations in *S. miltiorrhiza* [256].

As mentioned above, in *S. miltiorrhiza*, these PGRs solely influence the level of tanshinones and/or phenolic acids. In addition, crosstalk between these PGRs may also occur in other plants, suggesting that the interaction between these PGRs may be critical for the active compound production in *S. miltiorrhiza*. Moreover, in *S. miltiorrhiza*, some PGRs have an effect on the level of phenolic acids and tanshinones. However, investigation into the molecular mechanism behind this crosstalk is necessary. For example, GA_3_ has been shown to significantly improve the level of cryptotanshinone, and tanshinone IIA in the roots of the *HMGR4*-overexpression line, whereas IAA considerably inhibits tanshinone accumulation in the studied *S. miltiorrhiza* root material [257]. In *S. miltiorrhiza*, both MeJA and SA regulate the production and the transcription of tanshinone biosynthesis-genes in the hairy roots of *SmGGPPS*-overexpressing lines [167]. AUXs, CKs, and ABA enhance the production of cryptotanshinone, tanshinone I, and IIA in the hairy roots of *S. miltiorrhiza* [258].

Many kinds of hormones coexist in plants, which interact with each other to regulate the life activities of plants. Therefore, the crosstalk between PGRs is more important in the formation of active ingredients in *S. miltiorrhiza*. In addition, the production of tanshinones and phenolic acids is frequently induced by environmental factors, for example, light intensity and quality had a differential impact on the level of active compounds, red and blue light treatments significantly heighten the level of salvianolic acid B, and tanshinone content was more susceptible to light treatments than that of phenolic acids [259, 260]. Water deficit and salt stress induce the synthesis of cryptotanshinone, dihydrotanshinone I, tanshinone I, and tanshinone IIA in hairy roots of *S. miltiorrhiza* [148, 149, 171, 261]. As mentioned above, PGRs also regulate the level of tanshinones and phenolic acids in *S. miltiorrhiza*. The interaction of JA signaling and light influence the synthesis of cryptotanshinone, dihydrotanshinone I, tanshinone I, and tanshinone IIA by the SmHY5-SmBBX network [262]. Ultraviolet-B and MeJA working together improves the level of cryptotanshinone, tanshinone I, tanshinone IIA in hairy roots of *S. miltiorrhiza* [158]. As a result, the crosstalk between environmental stresses and PGRs are going to be the useful tool to increase the active compound production in *S. miltiorrhiza*. This is vital for the field cultivation and metabolic engineering of *S. miltiorrhiza*.

## Conclusions and perspectives

In conclusion, considerable advancements have been achieved in the study of the roles of PGRs on the production of active compounds in *S. miltiorrhiza*, but much work remains to be done. With the enhancement of people's health awareness, there is a growing market need for *S. miltiorrhiza*. However, *S. miltiorrhiza* faces significant challenges, such as varying levels of germplasm quality, degradation of varieties, and replanting diseases, all of which can significantly affect its quality. Therefore, there is an urgent need to enhance the production of the active compounds in *S. miltiorrhiza*. Secondary metabolism in plants is influenced by both endogenous and exogenous stimuli. PGRs are important factors that affect the plant secondary metabolite accumulation. Currently, PGRs are primarily used in horticultural plants, and their associated regulatory mechanisms have been elucidated. However, the application and regulatory mechanisms of PGRs in medicinal plants remain poorly understood. Based on the above findings, various PGRs exhibit distinct regulatory mechanisms for the active compounds of *S. miltiorrhiza*. In addition, different active compounds exhibit the same or varying responses to different PGRs. JAs, ABA, GAs, SA, and KARs simultaneously regulate the level of phenolic acids and tanshinones. PAs induce phenolic acids biosynthesis. CKs may act as positive elicitors of phenolic acid and tanshinone accumulation. Accumulation of tanshinones may be promoted by AUXs, ET, and NO. Recently, it was found that SL may promote tanshinones accumulation. It is yet unknown how much each signaling pathway contributes in relation to these PGRs in *S. miltiorrhiza*. Although the regulatory effects of several PGRs, such as MeJA, ABA, SA, and GAs, on the active compound production in *S. miltiorrhiza* have been comprehensively studied, the effects of AUXs, CKs, ET, NO, PAs, KARs, and SLs, particularly on BRs and MT are not well documented. There are few reports on the crosstalk between PGRs in *S. miltiorrhiza*, especially regarding the post-transcriptional regulation and epigenetic regulatory effects of PGRs on the active compound production in *S. miltiorrhiza*. Moreover, CKs, BRs, GAs, ABA, and SLs share common precursors with tanshinone and may have a significant impact on tanshinone accumulation of *S. miltiorrhiza*. Crosstalk between these PGRs in *S. miltiorrhiza* should also be considered. Furthermore, during the growing season, environmental stressors frequently have an effect on the quality and yield of *S. miltiorrhiza*. MeJA, SA, ABA, ET, NO, and PAs are important in how plants response to environmental stimuli. However, the roles and signal transduction mechanisms of these PGRs in response to environmental stress in *S. miltiorrhiza* remain unclear. Up to now, the use of PGRs for *S. miltiorrhiza* cultivation is rare. The application of certain PGRs in *S. miltiorrhiza* cultivated fields may increase yield and decrease active compound production. For example, chlormequat chloride (CCC), an inhibitor of GA synthesis, is often used in the cultivated fields of *S. miltiorrhiza* to increase the yield in many production areas. However, the tanshinone content is likely to significantly drop as a result of this procedure. Hence, further studies are needed on the selection of appropriate PGRs and their application in terms of tissue, stage, concentration, frequency, and methods. Moreover, the effect of the crosstalk between PGRs and the surrounding environment on the active compound biosynthesis in *S. miltiorrhiza* requires further investigation. Ensuring the safety of medication is crucial, and the presence of PGR residues in medicinal materials is of great importance. The role and regulatory mechanisms of PGRs in the production of active compounds in* S. miltiorrhiza* have extensive potential for guiding *S. miltiorrhiza* cultivation and metabolic engineering of this plant species.

## Data Availability

Not applicable.

## Abbreviations

AACT: Acetyl-CoA C-acetyltransferase
HMGR: Hydroxymethylglutaryl-CoA reductase
DXS: 1-Deoxy-d-xylulose 5-phosphate synthase
DXR: 1-Deoxy-d-xylulose5-phosphate reductoisomerase
IPP: Isopentenyl diphosphate
DMAPP: Dimethylallyl diphosphate
2OGD3: 2-Oxoglutarate-dependent dioxygenase 3
AOC: Alleneoxide cyclase
AOS: Allene oxide synthase
BRs: Brassinosteroids
cADPR: Cyclic ADP-ribose
CCC: Chlormequat chloride
CCD7: Carotenoid cleavage dioxygenase7
cGMP: Cyclic guanosine monophosphate
CKs: Cytokinins
CL: Carlactone
COI1: Coronatine insensitive 1
DEGs: Differentially expressed genes
ET: Ethylene
GID1: Gibberellin insensitive dwarf1
ICS: Isochorismate synthases
IPT: Isopentenyl transferase
JAs: Jasmonates
JAZ: Jasmonate ZIM-domain
JMT: Jasmonic acid carboxyl methyltransferase
KARs: Karrikins
KLs: KAR INSENSITIVE 2 (KAI2) ligands
LOG: Lonely guy
NAA: 1-Naphthaleneacetic acid
NO: Nitric oxide
OPR3: 12-Oxo-phytodienoic acid reductase 3
PAs: Polyamines
SA: Salicylic acid
SnRK2: SNF1-related protein kinase 2
TDZ: Thidiazuron
